# Implementing multi-component intervention to reduce antibiotic prescribing in primary care of rural China: a qualitative process evaluation of the trial

**DOI:** 10.1136/bmjopen-2025-108618

**Published:** 2026-01-16

**Authors:** Tingting Zhang, Xingrong Shen, Jing Chai, Rong Liu, Debin Wang, Lucy Yardley, Helen Lambert, Christie Cabral

**Affiliations:** 1 Centre for Academic Primary Care, Population Health Sciences, Bristol Medical School, University of Bristol, Bristol, UK; 2 School of Health Services Management, Ahui Medical University, Hefei, Anhui, China; 3 School of Psychology, University of Southampton, Southampton, UK; 4 School of Psychological Science, University of Bristol, Bristol, UK; 5 Population Health Sciences, Bristol Medical School, University of Bristol, Bristol, UK

**Keywords:** Antibiotics, China, Primary Care, Respiratory infections, QUALITATIVE RESEARCH

## Abstract

**Objectives:**

The overuse of antibiotics for respiratory tract infections in primary healthcare in rural China is a particular challenge and is highly related to antibiotic resistance. Our research team designed a multi-component intervention focusing predominantly on health practitioners to reduce antibiotic prescriptions in rural communities of China. The effects of the intervention were evaluated through a randomised controlled trial. This study was conducted alongside the trial to develop a contextualised understanding of the implementation of the intervention and related influencing factors.

**Design:**

Qualitative process study nested in a randomised controlled trial, including observation and semi-structured interviews.

**Setting:**

Primary healthcare in rural China.

**Participants:**

27 health practitioners from township health centres assigned to the intervention arm.

**Intervention:**

A complex intervention to reduce antibiotic prescriptions in rural communities of China, which includes the following components: training for health practitioners, a public letter of commitment, patient leaflets, a decision support system and a peer support group.

**Primary and secondary outcome measures:**

Not applicable.

**Analysis:**

Data were analysed using thematic analysis.

**Results:**

The overall multi-component intervention was described as useful in reducing antibiotic prescribing, with a particularly high acceptance and use of patient leaflets and the public letter of commitment among health practitioners. There were mixed views on the decision support system and peer support group. Practitioners reported usability-related barriers to using the decision support system during consultations. Practitioners did not understand the role or benefits of the peer support group and found it difficult to initiate group discussions, due to the lack of any existing clinical team at the primary care level.

**Conclusions:**

The multi-component intervention appears to be acceptable and useful in primary healthcare in rural China. Successful implementation requires a comprehensive understanding of the contextual characteristics of the setting. Interventions to reduce antibiotic prescribing in China in the future could consider wider stakeholders including patients, retail pharmacies and health authorities.

**Trial registration number:**

ISRCTN30652037 (01/12/2020).

**STRENGTHS AND LIMITATIONS OF THIS STUDY**

- The exploratory approach to data collection enabled us to capture both facilitators and barriers affecting the implementation of a complex intervention that has not been widely used in rural primary healthcare settings in China.
- Through recruiting practitioners from nearly all township health centres in the intervention arm and a diverse sample for follow-up interviews, our study provides a comprehensive understanding of the intervention’s implementation and its dynamic changes over time.
- A limitation of the study is the lack of direct patient accounts on the intervention due to the local outbreaks of COVID-19 in Anhui Province that changed the approval processes for fieldwork.
- Some participants in the follow-up interviews reported difficulties in recalling elements of the training booster due to the time elapsed.

## Introduction

 Antibiotic resistance (ABR), a serious threat to global public health and development, severely affects China.^1 2^ In 2019, there were an estimated 1.3 million infection-related deaths in China, accounting for 12.1% of total deaths in China, with nearly half of infection-related deaths associated with ABR and approximately 145 000 directly caused by it.^3^ The overuse of antibiotics especially for respiratory tract infections (RTIs) at the community level is highly related to rising antibiotic-resistant infections, such as resistant pneumonia.^4 5^ Between 2000 and 2010, antibiotic consumption increased by 36% across 17 countries, with 76% of this rise attributed to five countries, including China.^6^ Across China’s primary healthcare institutions, antibiotics were prescribed in over 50% of outpatient encounters,^7^ which was double the WHO’s recommended level of ≤30%.^8^

In China, antibiotic prescribing for RTIs in primary healthcare is guided by Guidelines for Primary Care of Acute Upper Respiratory Tract Infection (Practice Version 2018)^9^ and Guidelines for the Clinical Application of Antimicrobial Drugs (2015 Edition).^10^ For six types of RTIs that are commonly seen at the primary healthcare level, including acute upper RTI, acute tonsillitis, acute otitis media, acute sinusitis, acute bronchitis and pneumonia, antibiotics are generally not recommended except for pneumonia. However, antibiotics prescribed for various acute RTIs range from 55.1% to 68.5%.^11^ This is particularly challenging in rural areas of China, where multiple studies reported that the antibiotic prescribing rate for RTIs at the community level was around 85%,^12 13^ including one conducted in the study province.

There have been various interventions designed to reduce antibiotic prescribing for RTIs at the community level. However, most of them focused on high-income countries (HICs).^14 15^ Primary healthcare is a rapidly growing, but relatively weak, sector of China’s healthcare system that provides general medical services to residents at the community level.^16^ Township health centres (THCs) and, one level below them, village clinics are the primary healthcare institutions in rural China, with nearly 90% of patients choosing to visit these frontline health institutions as their first point of contact for outpatient care.^16 17^ Notably, China’s ‘primary health care’ system differs in important ways from that in high-income settings; in terms of quality, there are widespread gaps, particularly in rural areas.^18^ In THCs, approximately half of primary care practitioners’ education level was lower than junior medical college in 2018.^18^ Furthermore, interventions such as delayed prescribing that are found to be effective are not routine practices at the primary care level in rural China.^19^ Based on observational and ethnographic evidence from our previous project, we developed a complex multi-component intervention focusing predominantly on health practitioners as well as patients to reduce antibiotic prescriptions for RTIs in rural China. We used the Person-Based Approach, which entails extensive consultation and co-design with local practitioners and patients to develop a feasible and acceptable intervention.^20^ This complex intervention (see box 1) consists of five components, including (i) health practitioner training; (ii) public letter of commitment; (iii) patient leaflets; (iv) decision support system (DSS); and (v) peer support group. A cluster randomised controlled trial was then conducted to evaluate the effectiveness of the complex multi-component intervention on the antibiotic prescribing rate.^21^ The trial found the intervention was successful and results are reported in another paper.^22^

Box 1 **Details of five components of the complex intervention**

- Health practitioner training, covering: evidence of antibiotic resistance, current national guidelines for respiratory tract infections (RTIs) that recommend against antibiotic treatment for common RTIs seen in primary care and evidence of the overuse of antibiotics in rural China. The training was delivered by a locally respected senior clinician, supported by the research team.
- Public letter of commitment to reducing unnecessary antibiotic prescribing, designed and issued by the research team to health practitioners for sign-off and display in their clinics.
- Patient leaflets covering symptomatic treatment advice, safety-netting advice and an explanation for situations where antibiotics are not prescribed, aiming to provide practitioners with an alternative resource to give to patients when antibiotics are not prescribed and helping to reassure both patients and practitioners. The communication methods used were not specifically defined and were instead determined by doctors’ time constraints and personal preferences.
- Decision support system for health practitioners that summarises the national guidelines relevant to RTI diagnosis and treatment, reminding them that antibiotics are not recommended for most RTIs. This was provided in various formats: computer-based (a website) and paper-based version, and a shorter 1-page paper version generated based on feedback from initial interviews.
- Peer support group in which practitioners were asked to form a WeChat group with 2–5 others in the intervention to discuss challenges and seek support with changing antibiotic prescribing practice.

The qualitative process evaluation permits an assessment of the fidelity of the intervention implemented, a detailed description of the implementation processes and an understanding of the reach of intervention.^23^ It enables exploration of mechanisms of the intervention’s impact and potential effect modifiers and provides evidence for interpreting trial results.^23 24^ We therefore conducted a qualitative study nested within the trial, which aimed to investigate the views and experiences of health practitioners on the complex intervention during the process of implementation.

## Method

### Study design

The trial within which this study is embedded is a pragmatic two-armed trial randomised in 40 THCs in rural areas of Anhui Province, China. THCs, as well as village clinics sitting below the THC, are frontline health institutions of rural China’s healthcare system and are the first choice for most rural residents seeking outpatient care.^16 25^ The intervention was implemented in December 2021, with an intervention booster in June 2022 and the main trial recruitment was conducted between December 2021 and September 2022.

The qualitative process evaluation methods included observation and semi-structured interviews. The evaluation began immediately after the intervention was implemented and consisted of three phases:

- In phase one, we conducted an observation on the training event on 12 December 2021.
- In phase two, we interviewed practitioners who attended the training to obtain their views and experiences of using the intervention within 2 weeks of the training event.

The qualitative findings of observation and interviews were analysed to evaluate the implementation of the multi-component intervention, and based on the results, we developed an intervention booster and applied it in June 2022 to improve use of the intervention. The intervention booster included: additional evidence on the overuse of antibiotics in rural communities of China, a 1-page laminated poster summarised key RTI treatment advice from DSS, and support from the research team to facilitate the first meeting for peer support groups, where these had not been done.

- In phase three, we conducted follow-up interviews with a sub-sample of practitioners to understand their views and use of the intervention booster.

The Consolidated Criteria for Reporting Qualitative Research (COREQ) checklist was used to ensure comprehensive reporting of the study (see online supplemental file 1).

### Recruitment and data collection

#### Training event observation

A half-day training event, as one component of the complex intervention, was delivered on 12 December 2021 in Fuyang Municipality, Anhui Province. In total, 27 health practitioners attended the training, all of whom were selected from the THCs that were assigned to the intervention arm. This training event aimed to deliver antibiotic-related evidence and national guidelines on prescribing and to introduce the other four components of the intervention. It included four sessions and at the end of each session, we introduced one intervention component that was relevant to the content of that session and then arranged small group work for practitioners to discuss. Feedback to the whole group was then reported by each group in turn. Four iterations of this process took place focusing on topics of commitment letter, DSS, patient leaflet and peer support group, respectively.

We undertook observation on the training event to capture participants’ views, particularly questions and concerns, on antibiotic-related knowledge and the intervention. It was a hybrid observation due to COVID-19 international travel restrictions in Mainland China, with one researcher (RL) from China conducting in-person observation and another researcher (TZ) from the UK observing virtually using Tencent Meeting software. Both RL (MSc) and TZ (PhD) are trained and experienced female qualitative researchers.

The training event was video-recorded and field notes, particularly for whole group feedback, were made by both researchers using a pre-designed record sheet as guidance (see online supplemental file 2). Further, we pre-designed data collection forms for health practitioners to complete during the four group work discussions (see online supplemental file 3). We collected these forms at the end of training to help us capture information discussed in multiple groups at the same time that is challenging to observe.

#### In-depth semi-structured interviews

All 27 health practitioners who attended the training event were invited to participate in the initial semi-structured interviews. Two practitioners refused to participate, and one withdrew after the interview call was cut-off. Eight were then selected for the follow-up interviews, based on the variety in their experiences of using the multi-component intervention in their THCs. All eight selected practitioners agreed to participate in the follow-up interviews. Prior informed consent was obtained and participants received a leaflet informing them of the research aims and that the research was funded by the UK and Chinese governments and conducted by researchers from UK and Chinese government-funded universities.

Interviews were conducted by two researchers (XS and JC) in Mandarin. Both XS (PhD) and JC (PhD) are trained female qualitative researchers. Researchers were part of a large team who designed the intervention and implemented the trial. Practitioners were first contacted to confirm the time and then interviewed by phone or WeChat, in line with participant preference. The interviews were audio-recorded. The initial interviews were conducted between December 2021 and April 2022, and the follow-up interviews between December 2022 and February 2023. Interviews were guided by two topic guides exploring practitioners’ views and experiences of implementing the multi-component intervention and any recommendations to address barriers, and their views and experiences of using the intervention booster, respectively (see onlinesupplemental files 4  5).

### Data analysis

The video-recoding, field notes and data collection forms of the training event were analysed and summarised by TZ. All interviews were transcribed verbatim and thematically analysed in NVivo 12 Pro. A sub-sample of transcripts of initial interviews that captured varieties in practitioners’ views and practices was translated into English and two researchers (TZ and CC) independently read these bilingual transcripts and assigned initial codes to the data; views and practices that reoccurred within and across practitioners were allocated into conceptual categories and themes. After an initial coding framework was collaboratively developed, TZ indexed the remaining transcripts of initial and follow-up interviews based on the agreed framework and made refinements as necessary. Common codes and categories emerged across transcripts, indicating data saturation.

As the researchers collecting and analysing data were part of a larger team that designed the intervention and implemented the trial, there was awareness that pre-existing assumptions about antibiotic prescribing problems and beliefs in the value of behaviour change interventions might influence data collection and interpretation. To minimise potential bias, data were independently coded by multiple researchers, and findings were discussed regularly within the wider team, including those not directly involved in intervention design.

### Patient and public involvement

There was no formal involvement of patients or the public in this qualitative process evaluation.

## Results

Our qualitative evaluation consisted of a half-day training event observation with 27 attendees, 24 initial semi-structured interviews and eight follow-up interviews. Interviewed practitioners come from four THCs in Bozhou Municipality of Anhui Province and 13 THCs in Fuyang Municipality of Anhui Province. Table 1 shows participant characteristics. The results from observation and semi-structured interviews present key findings that emerged from the qualitative evaluation of the trial (see online supplemental file 6 for coding tree).

**Table 1:** Semi-structured interview participant characteristics

		Practitioner participants
**Total number**		24
Gender	Female	1
	Male	23
Age	<30	2
	30–39	2
	40–49	11
	50–59	8
	>=60	1
Education	Technical high school	4
	College	8
	Undergraduate	11
	Not available	1
Traditional Chinese Medicine background	Yes	15
	No	8
	Not available	1
Locations of THCs	Bozhou Municipality, Anhui Province	4
	Fuyang Municipality, Anhui Province	20
Length of working in current THCs	<10 years	9
	10–19 years	4
	20–29 years	6
	>=30 years	2
	Not available	3

THCs, Township health centres.

### Overall views on the complex intervention

Our interviews found that most practitioners are optimistic about the complex intervention, with the public letter of commitment and patient leaflets being seen as particularly useful. However, practitioners also believed that being a doctor working at the THC level, they already had antibiotic-related awareness and appropriate antibiotic prescribing practices:

*The commitment letter, we kept display it in our clinic so far, and it feels like an obligation, so this part has certain influences* [on reducing antibiotic prescribing]. [Practitioner 24, follow-up interview]

*The interventions are useful actually, but for me, for what education we have obtained, I always don’t prescribe inflammatory medicines [antibiotics] for these RTIs. … Yes, because of all these trainings, the professional development that we ever worked in high-level hospitals, so actually I seldomly used inflammatory medicines to them.* [Practitioner 21]

Practitioners therefore suggested expanding the coverage of the intervention to village clinics and considering the roles of retail pharmacies, pharmaceutical manufacturers and patients to intervene through methods like education and regulations. They also described the importance of the implementing process, where the local authority and government should take a leading role:

*I feel from grassroots level, from the level of village clinics to control and administrate would be best.* [Practitioner 9]

*Appropriate use of antibiotics, if you ask me, the first thing should be that the Ministry of Health gets involved in the management. The second thing is that in our location, especially the retail pharmacies need to have standardized management in order to achieve good results. Without this then all the training won’t achieve much, it won’t be that significant.* [Practitioner 20]

### Training: Mixed views on over-prescribing practices

The observation found that training went smoothly and practitioners were interested in the content. In the interviews, most practitioners believed that the training was useful in improving or confirming their understandings of antibiotics: do not prescribe antibiotics when they are not necessary.

However, some participants said that their beliefs and practices related to antibiotic prescribing will not change after the training since they have had such knowledge and appropriately prescribed antibiotics: “We don’t have many changes, we already know antibiotics should not be abused” [Practitioner 2].

There are mixed views on whether antibiotics are over-prescribed by practitioners. Some agreed but blamed patients, especially older rural residents, as practitioners believed they misunderstood antibiotics and reported that their demand for antibiotics led to overprescribing. Conversely, some practitioners reported that their workplaces have strictly regulated antibiotic use, with an estimated antibiotic prescription rate for RTI patients between 40% and 50% and that antibiotics are appropriately prescribed based on the evidence from blood tests or symptoms. Fewer antibiotics are prescribed at the community level than previously since efforts have been made over recent years and the overall culture has gradually improved.

*Definitely* [antibiotics are over prescribed], *rural residents’ current understandings are antibiotic treatment is needed as long as there is any illness and inflammation; even there is no inflammation, antibiotics should be used for prevention.* [Practitioner 7]

*The use of antibiotics has been much better than before, do not combine antibiotics, do not use antibiotics if not needed, like that.* [Practitioner 2]

Practitioners who had Traditional Chinese Medicine training felt that it is easier for them to use antibiotics rationally because Traditional Medicine treatment provides an alternative.

*… it is easier for me to change because I know Traditional Chinese Medicine. Sometimes you have a respiratory disease, and I simply use my traditional Chinese Medicine and the effect is very good.* [Practitioner 24]

### Commitment letter: Useful but only for young patients

From the observation, practitioners appeared to be happy with signing off and displaying the commitment letter. Consistent with these findings, all 24 practitioners reported in the interviews that they used the commitment letter, mainly by sticking it on the wall near the entrance of their clinics. Practitioners who obtained more than one copy put them in other places including the inpatient clinics, pharmacies or inpatient wards.

In the training event, practitioners expressed general concerns about reducing antibiotic prescribing related to patients, including their demand for antibiotics, the harm to the doctor-patient relationship when refusing patients’ requests, and the lack of understanding of the rationale of antibiotic use and limited communication. These concerns are particularly common in relation to the elderly who seek treatment for themselves or their grandchildren. Some practitioners reported concerns about limited access to essential medicines if antibiotic prescribing is restricted in their workplace.

No practitioners discussed the influence of the commitment letter on their own antibiotic prescribing practices, but viewed it as acting on patients differently according to age. Practitioners believed that only young people can understand the commitment letter: they had been reading the commitment letter and a few of them had discussed it with practitioners. Old people were not aware of the letter because of illiteracy, and practitioners have to explain to these patients why antibiotics should not be overused.

*Young people are okay* [in understanding the content of the commitment letter], *but older people are not; sometimes during the clinical* [consulting] *process, we should explain to these patients that antibiotics should be less used.* [Practitioner 10]

As a result, most practitioners believed that the commitment letter is useful for young patients due to their better understanding of antibiotics, while a few said it’s generally not useful since it lacks power: “How to say this one [commitment letter], it only has publicity function but is not very powerful” [Practitioner 4].

*…It’s more useful for young people but the old ones cannot understand it; they* [old people] *cannot accept this and only be satisfied once antibiotics are used*. [Practitioner 1]

To better educate the elderly, the role of grassroots facilities, such as village clinics, in disseminating information to community residents was highlighted. One practitioner suggested providing this commitment letter to village clinics as well to enable the coverage of different groups of patients.

*Not only the hospitals, like our township health centres, but also the village clinics that should be given few commitment letters, why? Because of the visit of patients, we see some parts of patients, and the village clinics also see some patients. So practitioners in the village practitioners can also disseminate it to patients, this* [village doctor] *is a very strong power.* [Practitioner 3]

### Patient leaflets: Variable utility according to age

Practitioners were found to be very familiar with using patient leaflets when this intervention was introduced in the training; they suggested additional ways to deliver the leaflets such as being given out by pharmacy staff, public health educating staff, or via WeChat. This intervention component was widely used and all practitioners reported that they had given leaflets to patients. Some practitioners also provided a brief explanation to patients while offering the leaflet. The content and detail of information practitioners provided depended on how busy they were. However, patients generally gave limited feedback, as practitioners said that patients did not ask any questions about the leaflets.

*When I was busy in the morning, with RTI patients, as we need to tick some parts of the form* [patient leaflet], *I will collect their information and quickly tell them this matter, and ask them to have a look after leaving here. But in the afternoon when I had more time, in most cases I will briefly explain the content of the leaflet to them and then ask them to take it home to have a look.* [Practitioner 9]

*I: Has any patient asked you any questions about the information from the leaflet? Or expressed any aspect that he did not understand? Is there such a thing?*

*R: No, he/she’s just listening. It would be fantastic if he/she could raise any question.* [Practitioner 18]

Three practitioners reported that they gave this leaflet to patients who came for other conditions because they wanted to educate the wider public. One gave leaflets to people who visit the THC to receive COVID-19 vaccination since he believed it is a faster way to send them out.

*Over those days when I came back from training we were just vaccinating COVID, you know, there were a lot of people came to get vaccinated, several hundreds. That’s very fast, very fast to send leaflets out.* [Practitioner 20]

Similar to the commitment letter, practitioners described the influences on patients rather than themselves and believed that the patient leaflet is mainly helpful to young people in terms of delivering antibiotic-related knowledge. Half of the interviewed practitioners (12/24) said that they would not give this leaflet to old or illiterate people, which diverges from the intended intervention. Practitioners believed that these people would not read the leaflet or treat it seriously. Further, they asserted that these people cannot understand and accept what the leaflet suggests even if practitioner explains it to them. Conversely, they considered that young people can understand the leaflet and are interested in it.

*Those people who took the leaflet are all young and, yes, they can understand it.* [Practitioner 6]

*For old people, we will not give them, since they will treat it like a supermarket’s leaflet and throw it away without any thinking. Well, we will only give* [leaflets to] *young patients.* [Practitioner 19]

One practitioner felt it was not very useful as patients would simply take the leaflet from practitioners without thinking carefully. One suggested that the content should be more straightforward and highlight the harms of overuse of antibiotics.

*I have sent them to patients. I feel it’s not very meaningful, they* [patients] *just say okay okay.* [Practitioner 20]

### Decision support system: multiple concerns and barriers to use

Most practitioners did use the computer-based DSS but only a few times just after the training. Despite the research team advising practitioners to use DSS in every RTI patient consultation, use was very varied. Some practitioners used it during less busy afternoons only, by recording patient information first and entering it into the DSS when they had spare time. One practitioner asked another junior practitioner to use the DSS for him since he was too busy. Four practitioners only included those RTI patients who were not prescribed antibiotics, and one practitioner chose only young patients who were likely to accept practitioners’ recommendations to include.

*Then I said that when I was busy, I first recorded the respiratory disease [patients] in the outpatient clinic, recorded their phone number, diagnosed and treated them, sent the leaflet to them, and when I was free, when there were not many things to do, then I would log on* [the system] *from the phone [to enter data], like this.* [Practitioner 19]

*The main reason is that their symptoms weren’t serious enough, so if they were given drugs it wouldn’t be any use* [of antibiotics]*, because they are likely to get better quickly anyway. The other reason is to do with age. Most of the elderly people won’t accept what you say even once you explain it to them*. [Practitioner 23]

There were multiple barriers related to the usability of DSS. Practitioners reported concerns about their capacity to use computer-based DSS during both the training event and interviews. Practitioners were extremely busy, particularly after local COVID-19 outbreaks. Also, they needed to use an existing electronic health records system during the consultation, which is a mandatory task required by the local authority. Using both systems doubled their consultation time and workload.

*I used the system at the beginning, but not now, because I am too busy and don’t have time to use it.* [Doctor 1]

*For example, when a patient visits and we need to work on the Zhiyi System, the information will be uploaded to our health authority and will be related to an examination. Now I still need to work on that thing* [DSS], *so I’ll ask the patient about his/her situation again and again.* [Doctor 11]

Some practitioners preferred to use the paper-based DSS so that they don’t need to switch between the two systems. Older practitioners reported that the paper-based one was much easier for them since they are not good at using computer. In addition, practitioners were concerned that patients would suspect that practitioners are playing on their computer/cell phone and not treating them seriously when using computer-based DSS, or that they are relying on computers rather than their professional knowledge to diagnose diseases.

*I’m typing very slowly on the computer, double vision, very slowly typing.* [Practitioner 3]

*I feel paper-based one is more convenient in practice, just put it on my desk and I can simply take one when working on this research, tick and work through it very quickly. If [I] work via cell phone or computer, patients will think you are playing computer but not treating them, right?* [Practitioner 11]

Practitioners also felt that the computer-based DSS contained too much information to work through in each consultation and was a poor fit with their own practice and experiences.

*Anyway that* [DSS] *wastes a lot of time, should be designed as simpler.* [Practitioner 16]

*The difference* [between using DSS to communicate with patients and normal communication] *is that sometimes DSS doesn’t have the symptoms that patients themselves reported*. [Practitioner 12]

In the follow-up interviews, we found most practitioners liked the revised DSS which was shortened to one page and paper-based; it was more frequently used during the consultation.

*Just put it* [paper-based DSS] *in the drawer. When we met suitable patients, we took it out and asked patients questions based on this form, and then recorded, and then publicised the use of antibiotics.* [Practitioner 22, follow-up interview]

There were mixed views on whether the content of the DSS was useful. While some practitioners felt the guidelines and safety netting advice were useful for guiding diagnosis and treatment and DSS can remind them to prescribe antibiotics rationally, some practitioners found that there is no improvement in doctor-patient communication and believed practitioners’ professional knowledge is more important. Practitioners also reported some misunderstandings, such as the belief that the computer-based DSS was designed to evaluate their practice or collect data for the research project.

*For myself, it is mainly because I feel that it* [DSS] *has a certain restraining effect, not use antibiotics when it doesn’t need to use, not to say that antibiotics can be over prescribed as before.* [Practitioner 1]

[The DSS is] *not useful, isn’t it used for your research aim? … you provided these* [diagnostic list and guideline] *to me, I have already had this knowledge, so they are not useful really.* [Practitioner 13]

### Peer support group: An unfamiliar intervention with various barriers

Practitioners had little to say about the use of peer support groups in the group work during the training. It was the least familiar intervention component for practitioners. We only identified four out of 24 practitioners (three from the same group and one from another group) who reported having conducted a peer support group discussion by April 2022. As part of the booster, our research team facilitated the first meeting for those groups who had not yet begun, after which most but not all practitioners started their own peer group.

Being busy was the most common reason mentioned by practitioners who did not start the discussion: they either generally didn’t have time or found it difficult to find a time when the whole group was available. Eight practitioners reported that they are unfamiliar with each other so felt embarrassed to start the discussion. In addition, there were a few concerns about topics. Practitioner 7 believed topics related to professional and technical skills are too sensitive to share with unfamiliar people. Although having conducted several meetings, practitioner 24 commented that he was not deeply involved in the meetings because topics were not closely relevant to his major in Traditional Chinese Medicine.

*…we’re all very busy and really cannot manage to make the discussion, you know…* [Practitioner 10]

These practitioners highlighted the important role of the group lead in scheduling and coordinating the meeting and said they would be happy to participate if the meeting was well organised.

*R: The group lead is very important, he/she needs to lead the group and arrange these people to do this and that, and requires that you must participate in the meeting every month.*

*I: If a group lead asks members to have a discussion, can you find a time?*

*R: Absolutely, I can ask for leave if needed, as long as he/she schedules a meeting time, such as every Monday or the last day of every month, we need to make sure the time in advance*. [Practitioner 3]

Some practitioners felt that there was no need to conduct this discussion. Three practitioners felt they didn’t have questions and treated the group discussion as unnecessary. One practitioner did not join the meeting for this reason even after the implementation of the intervention booster. One practitioner did not feel confident to discuss his performance, and one practitioner believed that practitioners would mainly rely on their own experience.

*Because I don’t feel I’ve done this thing [rational use of antibiotics] perfectly and, if I ask others, it may provide a sense of I’m doing better than others. So [I] will not ask others about how well they did.* [Practitioner 15]

*If people have done a job for many years, more than 20 years, it’s likely they have built up their own experiences. … You should make decision by yourself, and* [your practices] *will be same even after discussing with others …* [Practitioner 19]

Practitioners held different views on whether the peer support group is useful. Some said it is generally not useful except for improving communication skills. Some practitioners were more positive and felt it was a good place to share experiences and seek support. For example, practitioners working in different THCs could make comparisons between RTI patients and treatment in different areas.

*I think in practice, it doesn’t make much sense.* [Practitioner 11]

*If the group leader has treated an illness today, or seen a patient, or they want to talk about the symptoms, or if they’ve used a drug for a few days without improvement - everyone can talk about these things, because everyone’s experience is different, and everyone has different knowledge. Everyone can support one another.* [Doctor 20]

## Discussion

Our qualitative process evaluation provided insights into practitioners’ views and experiences of the intervention and the feasibility and acceptability of the different components. Health practitioners in THCs had an overall positive attitude towards the complex multi-component intervention and believed it was useful. They particularly liked patient leaflets and the public letter of commitment. They were also satisfied with the training, reporting that it confirmed their antibiotic-related knowledge and practices. There were mixed views about the DSS and peer support group and practitioners reported some barriers to implementation, such as a lack of time to meet up regularly and difficulty in using the DSS at the same time as their electronic health records system. They engaged with these two intervention components much better after intervention booster when the DSS was revised as a 1-page paper-based form and the peer support meeting was facilitated by the research team.

The mixed views on the peer support group highlight the challenge of adapting practices that have been effective elsewhere to a very different setting, even with the involvement of target populations at the intervention development stage. Peer support has been used successfully to improve health behaviour and outcomes, including a clinical team-based audit and feedback discussion as part of an effective intervention to reduce antibiotic prescriptions in hospitals in HICs.^26^,^28^ However, our study identified barriers when implementing peer support in primary care in rural China due to the lack of an existing clinical team at the THC level. Practitioners in THCs were not used to meeting and discussing clinical practice with their peers; they were confused about the aim and faced logistical difficulties in arranging the group meetings. They wanted a team lead to guide the process. A similar trial aimed at reducing antibiotic prescribing for RTI in child patients at primary care in rural China found that leadership by a respected senior doctor was linked to better performances in antibiotic prescribing.^29^ This may relate to the cultural dimensions described in Hofstede’s theory, which have been found to influence antibiotic use, particularly Power Distance, referring to the degree of authority and hierarchy that shape how decisions are made during medical encounters involving antibiotic prescribing. As hierarchy is deeply embedded in China’s healthcare system, practitioners may prefer leadership-driven decision-making rather than peer-based support.^30^ However, in the context of this trial, there were no senior doctors available to lead groups and no existing structures for sharing with or learning from peers. Most practitioners did not feel confident to lead a group or even share their practice with others, which may reflect their position working in lower-tier health centres, with limited biomedical education, and feelings of limited social capital and control.^31^

There were various barriers to THC practitioners using the computer-based DSS. Similar tools have been identified as important for improving healthcare practices and health quality.^32^ In the secondary care setting of HICs, studies reported reductions in antibiotic prescribing after shifting prescribing guidelines to digital-based design and positive attitudes towards these innovations among clinicians.^33 34^ Practitioners in our study reported perceived benefits of using the guideline and safety netting advice of DSS during diagnosis and communication, which covered key features of successful DSS in relation to improving clinical practices, including the provision of decision support specifically linked to case and provision of recommendations and communication rather than clinical assessment only.^32 35^ However, these successes of DSS were found to be largely context-dependent.^36^ In rural primary care, the roll-out of information technology (IT) systems for clinical care is patchy and functionally fragmented.^17^ In Anhui Province, there is a province-wide digital health platform, ‘Zhiyi’ system, developed by the Anhui Provincial Health Commission to enhance primary care, which is effectively required and has been widely implemented in most THCs and village clinics across the province to provide clinical decision support, electronic health record management and public health monitoring. As this system has been integrated into routine diagnosis and reporting processes, practitioners were concerned that the conflict with DSS would increase their workload and reduce work efficiency. Practitioners, particularly older ones, would need to spend extra time and effort to get used to the new computer-based system. Similar usability issues that limit engagement have been found in other primary care settings.^37^ The causes of the additional workload that was the key barrier to use were all addressed by the creation of a 1-page paper summary of the key guidance.

Practitioners liked and used the public letter of commitment and patient leaflets. The written information used during consultation can enhance doctor-patient interaction and patients’ limited health literacy, encourage self-management and help to increase prescribers’ confidence in patient safety and satisfaction when antibiotics are not prescribed.^38 39^ Our trial data suggested that in total, 61.1% of patients visiting intervention arm THCs received a leaflet, 55.6% read it and 42.4% found it easy to understand.^22^ The effectiveness of patient-facing materials in reducing antibiotic prescriptions for infectious diseases has been identified by multiple studies.^38 39^ Practitioners liked these elements of the interventions because they believed that antibiotic overuse is driven primarily by patients and the letter and leaflet were tools for practitioners to educate and manage patients, most of them felt confident that, as doctors working at the THC level, they already adhered to appropriate antibiotic prescribing practices. It is worth noting that blood tests for patients presenting with RTIs are available at the THC level, and practitioners often interpret a high white blood cell count as an indication for antibiotic use. However, this interpretation is inaccurate, as an elevated white blood cell count does not reliably distinguish between bacterial and viral infections or other causes of inflammation. Our training has recommended conducting C-reactive protein testing along with a blood test, which was intended to reduce diagnostic uncertainty.

However, practitioners had different attitudes and practices with regard to these intervention components related to patient age. In line with findings of interviews with caregivers in a similar trial in rural areas of China, practitioner interviewees felt that leaflets were less useful with people with low literacy and educational level, such as the grandparents of an ill child.^29^ A systematic review focusing on China has reported that patients and caregivers who are older, have lower education levels or live in rural areas tend to have higher expectations for antibiotic prescriptions, which is associated with an increased risk of inappropriate prescribing.^40^ As older people in rural areas are generally characterised by lower levels of education, there is a widespread perception that this group is highly likely to demand antibiotic prescriptions and is more difficult to communicate with or persuade. Furthermore, in China, infections are commonly referred to as ‘inflammation’ (yan in Mandarin) and antibiotics as ‘anti-inflammatory medicines’ (xiaoyan yao in Mandarin) by patients and practitioners when talking to patients, while the Mandarin terms for ‘infection’ and ‘antibiotics’ are unknown to most of the patients. As inflammation is more readily connected by Chinese patients with a ‘hot’ condition in body in traditional Chinese medicine perspective with symptoms of redness and heat, this language reflects a more fundamental issue that the way Chinese people often understand infections as a bodily reaction rather than an invasion by external pathogens, and antibiotics as anti-inflammatory agents that ‘cool’ this bodily reaction.^41^ This suggests a different illness model among patients, which may have been more prevalent among older patients, from the biomedical model that conceptualises infections in terms of microbes. Consequently, biomedical messages such as ‘antibiotics don’t work on viruses’ may be less meaningful and persuasive to them, particularly for older people.

Although our patient leaflet and letter of commitment were translated into local terminology and incorporated images to develop a narrative to support communication, the advice was framed in relation to the biomedical model, which may have made it less comprehensible for older patients. Another study reported that showing a video in the waiting areas was easier to understand and more useful, suggesting a potentially effective way to deliver information to old patients that could be used to influence patient consulting and practitioner antibiotic prescribing decisions.^29^ However, the feasibility of this approach would depend on the financial resources and facilities available in rural institutions. Future interventions should consider incorporating culturally sensitive, visually and narratively based forms of communication to improve accessibility among older people.

### Strengths and Limitations

Our study recruited practitioners from almost all THCs in the intervention arm for initial interviews and a diverse sample for follow-up interviews, enabling all aspects of the intervention to be investigated. A key study limitation is that no interviews were conducted with patients, so we do not have patient views of the intervention, we only have practitioners’ reports of patients’ reactions to the intervention. We were planning to conduct more observations in the clinics during the implementation of the intervention but were not able to do this due to the outbreaks of COVID-19 in Anhui Province, restrictions on movement and changes to approval processes for fieldwork. Some participants in the follow-up interviews reported difficulties in recalling elements of the training booster due to the time elapsed.

## Conclusions

Our qualitative study provided insights into practitioners’ views and experiences of the multi-component intervention and each individual component to reduce antibiotic prescribing in primary care in rural China. The overall intervention was reported as useful; however, there were particular barriers and concerns about using computer-based DSS and the peer support group component was not fully implemented, due to contextual characteristics of China’s rural primary care system. This also suggests potential difficulties when transferring interventions developed and implemented in European countries into low and middle-income settings such as China. Future interventions to reduce antibiotic prescribing in China should further consider context-specific factors and include wider stakeholders at the system level such as patients, retail pharmacies and health authorities.

## Supplementary material

10.1136/bmjopen-2025-108618 online supplemental file 1

10.1136/bmjopen-2025-108618 online supplemental file 2

10.1136/bmjopen-2025-108618 online supplemental file 3

10.1136/bmjopen-2025-108618 online supplemental file 4

10.1136/bmjopen-2025-108618 online supplemental file 5

10.1136/bmjopen-2025-108618 online supplemental file 6

## Data Availability

Data are available upon reasonable request.

## References

[1] O’Neill J (2016). Tackling drug-resistant infections globally: final report and recommendations.

[2] Qu J, Huang Y, Lv X (2019). Crisis of Antimicrobial Resistance in China: Now and the Future. Front Microbiol.

[3] Zhang C, Fu X, Liu Y (2024). Burden of infectious diseases and bacterial antimicrobial resistance in China: a systematic analysis for the global burden of disease study 2019. *Lancet Reg Health West Pac*.

[4] Teng CL (2014). Antibiotic prescribing for upper respiratory tract infections in the Asia-Pacific region: A brief review. Malays Fam Physician.

[5] Zhuo C, Wei X, Zhang Z (2020). An antibiotic stewardship programme to reduce inappropriate antibiotic prescribing for acute respiratory infections in rural Chinese primary care facilities: study protocol for a clustered randomised controlled trial. Trials.

[6] Van Boeckel TP, Gandra S, Ashok A (2014). Global antibiotic consumption 2000 to 2010: an analysis of national pharmaceutical sales data. Lancet Infect Dis.

[7] Yin X, Song F, Gong Y (2013). A systematic review of antibiotic utilization in China. J Antimicrob Chemother.

[8] World Health Organisation Using indicators to measure country pharmaceutical situations: fact book on who level 1 and level 11 monitoring indicators.

[9] Chinese Medical Association (2019). Guideline for primary care of acute upper respiratory tract infection: practice version(2018). Chin J Gen Pract.

[10] (2015). Guidelines for the clinical application of antimicrobial drugs (2015 edition).

[11] Fu M, Gong Z, Li C (2023). Appropriate use of antibiotics for acute respiratory infections at primary healthcare facilities in China: a nationwide cross-sectional study from 2017 to 2019. Lancet Reg Health West Pac.

[12] Jiang Q, Yu BN, Ying G (2012). Outpatient prescription practices in rural township health centers in Sichuan Province, China. BMC Health Serv Res.

[13] Chai J, Coope C, Cheng J (2019). Cross-sectional study of the use of antimicrobials following common infections by rural residents in Anhui, China. BMJ Open.

[14] Huttner B, Goossens H, Verheij T (2010). Characteristics and outcomes of public campaigns aimed at improving the use of antibiotics in outpatients in high-income countries. Lancet Infect Dis.

[15] Andrews T, Thompson M, Buckley DI (2012). Interventions to influence consulting and antibiotic use for acute respiratory tract infections in children: a systematic review and meta-analysis. PLoS One.

[16] World Health Organisation (2015). Regional office for the western p. people’s republic of china health system review.

[17] Li X, Lu J, Hu S (2017). The primary health-care system in China. Lancet.

[18] Li X, Krumholz HM, Yip W (2020). Quality of primary health care in China: challenges and recommendations. Lancet.

[19] Spurling GKP, Del Mar CB, Dooley L (2013). Delayed antibiotics for respiratory infections. Cochrane Database Syst Rev.

[20] Yardley L, Morrison L, Bradbury K (2015). The person-based approach to intervention development: application to digital health-related behavior change interventions. J Med Internet Res.

[21] Cong W, Chai J, Zhao L (2022). Cluster randomised controlled trial to assess a tailored intervention to reduce antibiotic prescribing in rural China: study protocol. BMJ Open.

[22] Xingrong Shen BS, Cui E, Liu R Debin wang a complex intervention to reduce antibiotic prescribing in rural china: a cluster randomised controlled trial. The Lancet Regional Health– Western Pacific.

[23] Moore GF, Audrey S, Barker M (2015). Process evaluation of complex interventions: Medical Research Council guidance. BMJ.

[24] Grimshaw JM, Zwarenstein M, Tetroe JM (2007). Looking inside the black box: a theory-based process evaluation alongside a randomised controlled trial of printed educational materials (the Ontario printed educational message, OPEM) to improve referral and prescribing practices in primary care in Ontario, Canada. Implementation Sci.

[25] National Health Commission (2019). 2019 annual china health statistics.

[26] Santillo M, Sivyer K, Krusche A (2019). Intervention planning for Antibiotic Review Kit (ARK): a digital and behavioural intervention to safely review and reduce antibiotic prescriptions in acute and general medicine. J Antimicrob Chemother.

[27] Cross ELA, Sivyer K, Islam J (2019). Adaptation and implementation of the ARK (Antibiotic Review Kit) intervention to safely and substantially reduce antibiotic use in hospitals: a feasibility study. J Hosp Infect.

[28] Llewelyn MJ, Budgell EP, Laskawiec-Szkonter M (2023). Antibiotic review kit for hospitals (ARK-Hospital): a stepped-wedge cluster-randomised controlled trial. Lancet Infect Dis.

[29] Wei X, Deng S, Haldane V (2020). Understanding factors influencing antibiotic prescribing behaviour in rural China: a qualitative process evaluation of a cluster randomized controlled trial. J Health Serv Res Policy.

[30] Deschepper R, Grigoryan L, Lundborg CS (2008). Are cultural dimensions relevant for explaining cross-national differences in antibiotic use in Europe?. BMC Health Serv Res.

[31] Chen M, Kadetz P, Cabral C (2020). Prescribing Antibiotics in Rural China: The Influence of Capital on Clinical Realities. *Front Sociol*.

[32] Thursky K (2006). Use of computerized decision support systems to improve antibiotic prescribing. Expert Rev Anti Infect Ther.

[33] Westphal J-F, Jehl F, Javelot H (2011). Enhanced physician adherence to antibiotic use guidelines through increased availability of guidelines at the time of drug ordering in hospital setting. Pharmacoepidemiol Drug Saf.

[34] Charani E, Kyratsis Y, Lawson W (2013). An analysis of the development and implementation of a smartphone application for the delivery of antimicrobial prescribing policy: lessons learnt. J Antimicrob Chemother.

[35] Kawamoto K, Houlihan CA, Balas EA (2005). Improving clinical practice using clinical decision support systems: a systematic review of trials to identify features critical to success. BMJ.

[36] Pinder R, Berry D, Sallis A (2015). Antibiotic prescribing and behaviour change in healthcare settings: literature review and behavioural analysis.

[37] Rawson TM, Moore LSP, Hernandez B (2017). A systematic review of clinical decision support systems for antimicrobial management: are we failing to investigate these interventions appropriately?. Clin Microbiol Infect.

[38] de Bont EGPM, Alink M, Falkenberg FCJ (2015). Patient information leaflets to reduce antibiotic use and reconsultation rates in general practice: a systematic review. BMJ Open.

[39] Moerenhout T, Borgermans L, Schol S (2013). Patient health information materials in waiting rooms of family physicians: do patients care?. Patient Prefer Adherence.

[40] Lin L, Sun R, Yao T (2020). Factors influencing inappropriate use of antibiotics in outpatient and community settings in China: a mixed-methods systematic review. BMJ Glob Health.

[41] Lambert H, Chen M, Cabral C (2019). Antimicrobial resistance, inflammatory responses: a comparative analysis of pathogenicities, knowledge hybrids and the semantics of antibiotic use. Palgrave Commun.

