# Supplementary material for: Implementing multi-component intervention to reduce antibiotic prescribing in primary care of rural China: a qualitative process evaluation of the trial
Source: BMJ Open. 2026 Jan 16;16(1):e108618. doi: 10.1136/bmjopen-2025-108618 (PMC12815065; doi:10.1136/bmjopen-2025-108618)
Supplement: online supplemental file 2 [file bmjopen-16-1-s002.docx]

**Record Sheet**

This form should be filled by a training assistant from AMU at the registration period and during the training.

| **Date:** | | |
| --- | --- | --- |
| **Location:** ______township, ________county/district, ______municipality | | |
| **Basic information of training** | | |
| Training start time | |  |
| Training end time | |  |
| Presenter/s | |  |
| Training assistants and their responsibilities | |  |
| Please give a description of the training place (e.g. is it in a hotel or a classroom? Spacious? Comfortable? Enough space for group work? other significant characteristics?) | |  |
| **Basic information of participants** | | |
| Township health centre name | | How mang hours did you take for today’s journey? |
|  | |  |
|  | |  |
|  | |  |
|  | |  |
|  | |  |
|  | |  |
|  | |  |
|  | |  |
|  | |  |
| If there is anyone else participating, please note: | | |
| **Were there any changes or additions to the planned training (to content, timing, order)? If so please describe here**: | | |
| **Please record all questions being asked by participants during whole class sessions:** | | |
| Part 1 | [expand form as needed] | |
| Part 2 |  | |
| Part 3 |  | |
| Part 4 |  | |
