# Supplementary material for: Implementing multi-component intervention to reduce antibiotic prescribing in primary care of rural China: a qualitative process evaluation of the trial
Source: BMJ Open. 2026 Jan 16;16(1):e108618. doi: 10.1136/bmjopen-2025-108618 (PMC12815065; doi:10.1136/bmjopen-2025-108618)
Supplement: online supplemental file 3 [file bmjopen-16-1-s003.docx]

**Form 1 – work group and discission on letter of commitment**

**Please fill your Township Health Centre name (all group members need to fill):**

**Please nominate a leader in your group who will be responsible for making brief notes on your discussion on below questions. You can then use these notes in your presentation in next session (whole group feedback), and please give this form back to one of the training assistants after the whole group feedback session.**

1. How will doctors use or display letter of commitment in their clinic?
2. Any questions, concerns or barriers to using this letter of commitment?
3. How could any concerns or barriers be overcome?

**Form 2 – work group and discission on DSS**

**Please fill your Township Health Centre name (all group members need to fill):**

**Please try out DSS on both your own laptops and on paper using case studies provided in page 2. Then nominate a leader in your group who will be responsible for making brief notes on your discussion on below questions. You can use these notes in your presentation in next session (whole group feedback), and please give this form back to one of the training assistants after the whole group feedback session.**

1. How will DSS work in practice?
2. Any questions, concerns or barriers to using DSS?
3. How could any concerns or barriers be overcome?
4. Which format is preferred (computer or paper)?

**Form 3 – work group and discission on patient leaflet**

**Please fill your Township Health Centre name (all group members need to fill):**

**Please nominate a leader in your group who will be responsible for making brief notes on your discussion on below questions. You can then use these notes in your presentation in next session (whole group feedback), and please give this form back to one of the training assistants after the whole group feedback session.**

1. How will doctors use patient leaflet with patients?
2. Any questions, concerns or barriers to using this patient leaflet?
3. How could any concerns or barriers be overcome?

**Form 4 – work group and discission on peer support group**

**Please fill your Township Health Centre name (all group members need to fill):**

**The elected leader of WeChat group will be responsible for making brief notes on your discussion on below questions. You can then use these notes in your presentation in next session (whole group feedback), and please give this form back to one of the training assistants after the whole group feedback session.**

1. Each group member’s presentation on one case study (see page 2 for case studies)

Member 1:

Member 2:

Member 3:

**…**

1. How to overcome any challenges?
2. How to make this peer support group work in their clinic?

**Case studies 案例研讨**

Case study 1（案例一）:

Miss Zhao is a 15 year-old female presenting to your township clinic complaining of sore throat and sensitivity to cold, began approximately 2 days ago. Her body temperature is 38.2 degree, with swelling and increased blood flow of the tonsils. Lymphatic nodes are swelling with pressure-sensitive pain. What do you do for her?

赵小姐是一名 15 岁的女性，因从两天前开始喉咙痛和畏寒而来您的门诊就诊。她的体温为 38.2 度，扁桃体充血肿大，颈部淋巴结肿大并伴有压痛。问题：你打算怎么做？

Case study 2（案例二）:

Mr Liu, a 37-year-old man, comes to your township clinic complaining of nasal obstruction accompanied by facial pain that has persisted for the past week. What do you do?

刘先生，37 岁，来到您的门诊就诊。主诉在过去一周持续鼻塞并伴有面部疼痛。问题：你打算怎么做？

Case study 3（案例三）:

A man in his late 50s presented with an acute cough. He had been feeling sick for the past two weeks. He produces green sputum and sometimes experiences shortness of breath, for example when riding his bicycle. He is asking for an antibiotic, because a neighbour who was coughing for a few weeks turned out to have a pneumonia. On examination, his pharynx is very red and body temperature is 37.8 degree. Auscultation of the lungs reveals some wheezes. What do you do?

一名 50 多岁的男子因咳嗽前来就诊。在过去的 两周里他一直感觉不舒服。咳嗽时有黄绿色痰，并且有时，比如骑自行车时，会出现呼吸急促。他向你提出开消炎药的要求，因为他的一位邻居因为咳嗽了几个星期最后得了肺炎。查体时，他的咽部非常红，体温为37.8度。肺部听诊可发现一些哮鸣音。问题：你打算怎么做？
