# Supplementary material for: Implementing multi-component intervention to reduce antibiotic prescribing in primary care of rural China: a qualitative process evaluation of the trial
Source: BMJ Open. 2026 Jan 16;16(1):e108618. doi: 10.1136/bmjopen-2025-108618 (PMC12815065; doi:10.1136/bmjopen-2025-108618)
Supplement: online supplemental file 4 [file bmjopen-16-1-s004.docx]

**Interview topic guide:**

1. What do you feel about the training evidence and content?

- Did the training change how you think about antibiotics? What are changed?
- Do you agree that antibiotics are over prescribed?

Do you feel the training content works in your clinics?

Do you feel there will be any particular facilitators or barriers?

- If doctors do not follow the evidence for certain patients (e.g. older), why? *[Try to get doctor to get their own answer first, then if not mentioned, probe to see if they have concerns such as loosing patients, or whether they feel antibiotic control is less important in certain groups, or whether doctors believe AMR is relevant to individual person rather than bacteria]*
- Some doctors reported in the training that they need support, including financial support, from local authorities to engage in these guideline and activities, what do you think about this?
  - What financial arrangements are likely to result in you prescribing fewer antibiotics?
  - Is there any mechanism for this already?

1. How do you use the letter of commitment in your clinic?

Do you feel this letter give some information to your patients?

- Do your patients read it or interest in it? Do they ask you questions about this letter?
- Can they understand it? If not, where are the difficult parts?

Do you think this is useful?

1. Do you use the DSS?

If yes

- When do you usually use it (e.g. during the consultation? During less busy time?)?
- How do you feel about the computer DSS and paper based DSS?
  - How often do you use computer DSS and/or paper based DSS?
  - Do they work with multiple patients in your clinic?
- What data do you enter?

If not

- what are the barriers?
- What would make it easier to do?

What do you think it the purpose of the DSS?

Do you ever find the advice from the DSS is different to what you did in clinic?

Is DDS practical for your patients?

- Do you feel the list of diagnosis appropriate to your patients?
- Do you feel the guideline on RTIs right for your patients?
- Do you feel the safety netting advice useful for your patients? Will it work in your clinic?

1. How do you usually use this patient leaflet with your patients?

- How often do you use it?
- Do you feel it give right information for your patients?
- Do you feel your patients can understand it? If not, where are the difficult parts?

1. Have you managed to do this peer support group?

If not

- what are the barriers?
- What would make it easier to do?
- Do you think this peer support group will be useful?

If yes,

- how did you do this?
- How did you find a time for the group discussion?
- Is there any barrier to lead/participate in the peer support group discussion?
- Do you think this is useful?

*[Interviewer can encourage doctor to engage in this intervention like saying you can find a peer to talk to privately if you find too difficult to talk in the group, or if the lead does not organise the meeting*

*Interviewer can also encourage the lead of group to schedule a meeting]*

1. Do you have any recommendations on how we can address barriers or concerns?
2. Background information:

- Age
- Educational background:
- Highest education certificate
- Any TCM background?
- How long have your biomedical and/or TCM training lasted?
- How long have you been working in this THC?
- Which THC are you working in?
- Where is this THC located?
