# Supplementary material for: Implementing multi-component intervention to reduce antibiotic prescribing in primary care of rural China: a qualitative process evaluation of the trial
Source: BMJ Open. 2026 Jan 16;16(1):e108618. doi: 10.1136/bmjopen-2025-108618 (PMC12815065; doi:10.1136/bmjopen-2025-108618)
Supplement: online supplemental file 5 [file bmjopen-16-1-s005.docx]

**Follow-up interview topic guide:**

1. What do you remember about the second (refresher) training?

- Was it useful (if yes, in what way)?
- Did you learn anything new (if yes, what)?
- How do you feel the training content works in your clinics?

1. What do you think of the one-page laminated DSS (national guideline)?

- Is it useful?
- Is the information correct?

2.1 Do you use the laminated DSS?

If yes

- Can you give more details about how do you usually use it? (e.g. where do you put it? When do you use it? how often do you use it?)
- Do you ever find the advice from the laminated DSS is different to what you did in clinic? (if yes, in what way is it different? Do you ever follow the DSS advice instead of usual practice?)

If not

- What are the barriers to use?

1. Have you managed to do the peer support group or to discuss in pairs?

If yes,

- Please describe the group / pair (who set it up, how many doctors were there, who led the discussion, was there facilitation by AMU?)
- What is useful (if yes, in what way)

If not,

- What are the barriers?

1. Do you think the intervention helped reduce antibiotic use?

If yes,

- which part of the intervention do you think helped reduce antibiotic use?
- Were any parts less useful?

If no,

- Why not?
- What would help reduce antibiotic use?

1. Background information (only if we have not interviewed this doctor before):

- Age
- Educational background:
- Highest education certificate
- Any TCM background?
- How long have your biomedical and/or TCM training lasted?
- How long have you been working in this THC?
- Which THC are you working in?
- Where is this THC located?

If we have interviewed this doctor before, give id number from previous interview:
