# Supplementary material for: Implementing multi-component intervention to reduce antibiotic prescribing in primary care of rural China: a qualitative process evaluation of the trial
Source: BMJ Open. 2026 Jan 16;16(1):e108618. doi: 10.1136/bmjopen-2025-108618 (PMC12815065; doi:10.1136/bmjopen-2025-108618)
Supplement: online supplemental file 6 [file bmjopen-16-1-s006.docx]

Qualitative process evaluation coding tree

| Name | Description |
| --- | --- |
| **Influences** |  |
| **Contextual or external influences** |  |
| Inappropriate use by peers | Pressures from the fact that antibiotics are inappropriately used by other doctors and retail pharmacies |
| Other influences, e.g. organisational |  |
| - Institutional characteristics | Busy; Crowded with patients, lack of tests, less strictly regulated when compared with higher-level hospitals; Patients’ higher expectation on being well treated, including being prescribed antibiotics, when compared with village clinics |
| - official or financial support | The importance of support from the health authority level or financial support, including insurance |
| - pandemic control |  |
| **Doctors’ views and experiences of patients** |  |
| - Loss of patients | Concern about losing patients when not prescribing antibiotics |
| - Patient characteristics | High quality (sensible patients) vs low quality (anxious patients); Child patients; Patients’ beliefs; Communication |
| - Patient expectations and demand | Patients expected to be well treated in this level of institutions - non-antibiotics (e.g. drink more water only) cannot meet their expectations; Some patients will demand antibiotics |
| - Trust relationship | Difficult to use intervention when consulting with patients who don’t trust doctors; concerns about not prescribing antibiotics will undermine patients’ trust in doctors |
| **Doctors’ illness model vs biomedical model** |  |
| - Severity of illness | Antibiotic prescription is based on the patients’ health condition; Considering the time needed for recovery |
| - Blood test | The golden criteria |
| - Type of RTIs |  |
| **Doctors’ views and experiences on interventions** |  |
| **Overall views on interventions** |  |
| - Knowledge or attitudes towards antibiotic usage | Doctors’ understandings of the overuse of antibiotics and the importance of avoiding the overuse of antibiotics |
| - What has been done | Doctors already have the awareness, or what has been done appropriately |
| - Feedback on intervention & booster | Positive & negative; further suggestions |
| - Misunderstanding of interventions | e.g. misunderstandings on how to use DSS, which types of patients should be included |
| - Personal characteristics | e.g. younger doctors vs older doctors |
| - Understanding of AMR |  |
| **Commitment letter** |  |
| - Feedback on the commitment letter | Useful or not |
| - Feedback from patients | Old vs young patients – e.g. older patients have no communication with doctors |
| - Experiences of using the commitment letter |  |
| **DSS** |  |
| - Experiences of using DSS | Selecting patients to be included; filling info at a less busy afternoon time |
| - Overall feedback on the DSS & booster | Useful or not |
| - Main problems |  |
| - - Diagnostic system overlapping | Doctors already have one compulsory online system that they need to use during consultations |
| - - Access methods | DSS can be accessed and work via cell phone as well, which doctors believe will cause patients’ concerns and complaints |
| - - Other issues | Too long and detailed; listed drugs are not available in THCs |
| **Patient leaflets** |  |
| - Experiences of using leaflets | Selecting patients |
| - Feedback on the leaflets | Useful or not |
| - Patient type & illiteracy problem | e.g. Old patients cannot read the patient leaflets |
| **Peer support group** |  |
| - Experiences of using peer support |  |
| - Feedback on the peer support & booster | Useful or not |
| - Main problems |  |
| - - Prefer own experience | Doctors trust their own experience and don’t want to discuss with others in a peer support group |
| - - Difficult to facilitate the meeting | Suitable time for everyone; The role of group lead |
| - - Feel shy and not good at communicating |  |
