# Supplementary material for: Implementing multi-component intervention to reduce antibiotic prescribing in primary care of rural China: a qualitative process evaluation of the trial
Source: BMJ Open. 2026 Jan 16;16(1):e108618. doi: 10.1136/bmjopen-2025-108618 (PMC12815065; doi:10.1136/bmjopen-2025-108618)
Supplement: Supplementary file 7 [file bmjopen-2025-108618.reviewer_comments.pdf]

PEER REVIEW HISTORY

BMJ Open publishes all reviews undertaken for accepted manuscripts. Reviewers are asked to complete a checklist review form and are provided with free text boxes to elaborate on their assessment. These free text comments are reproduced below.

ARTICLE DETAILS

Title (Provisional)

Implementing a multi-component intervention to reduce antibiotic prescribing in primary care in rural China: a qualitative process evaluation of a trial

Authors

Zhang, Tingting; Shen, Xingrong; Chai, Jing; Liu, Rong; Wang, Debin; Yardley, Lucy; Lambert, Helen; Cabral, Christie

---

VERSION 1 - REVIEW

**Reviewer**	**1**
**Name**	**Llor, Carl**
**Affiliation**	**University of Southern Denmark, Department of Public Health, General Practice.**
**Date**	**08-Sep-2025**
**COI**	**None**

The study is both interesting and relevant. However, I would kindly request the authors to provide more context and explanation regarding the Chinese healthcare system. From a Western perspective, some aspects of the article are difficult to fully understand due to differences in clinical practice and healthcare infrastructure. For instance, it would be helpful to clarify what the "Zhiyi system" refers to, as this term may not be familiar to international readers. Additionally, including information about Chinese clinical guidelines for the management of RTIs would significantly enhance the article’s accessibility. Even a brief overview in the supplementary material would be valuable, particularly for common RTIs such as pneumonia, acute exacerbations of COPD, acute bronchitis, pharyngitis, and tonsillitis, since most readers may not be acquainted with the Chinese recommendations for these conditions. Moreover, the concept of using "anti-inflammatory medicines" in place of antibiotics may appear unusual or even concerning to those trained in Western medicine. Further clarification and discussion of this practice, especially regarding its rationale, usage, and supporting evidence in China, would be important for international understanding and comparison.

In the Strengths and Limitations sections (both), you mention that a limitation of the study is the lack of direct patient accounts regarding the intervention. However, you also state that patients' views and responses were indirectly captured through practitioner interviews and informal conversations with fieldworkers from both intervention and control clinics. This remains a supposition; conclusions about patients' perspectives cannot be reliably drawn without directly interviewing the patients themselves. I kindly suggest deleting this latter part for accuracy.

Could you please clarify who issued the public letter of commitment to reducing unnecessary antibiotic prescribing, which was provided to health practitioners for sign-off and display in their clinics? Was it issued by local or national health authorities, or by the steering committee of the randomised clinical trial? Additional details would be helpful. I suggest including this information in Box 1 for clarity.

Regarding the distribution of leaflets, could you please provide more detail on how doctors in the intervention group actually used them? Specifically, was the process limited to simply handing out the leaflets, or did it also involve discussing key points with patients during the consultation? This is an important consideration, as the effectiveness of the intervention largely depends on how the information is communicated and received by patients. As some doctors rightly pointed out, handing out a leaflet alone is of limited value if time is not spent addressing the conflicting information and guiding patients through it.

Did you have any discussions with doctors about the individual components of the multifaceted intervention? It would have been valuable to engage with frontline doctors beforehand to assess the perceived relevance, feasibility, and acceptability of each of the five components. Based on the information provided in the manuscript, it appears that few doctors actively used the decision support system or participated in the peer support groups. Further explanation of why this was the case, and whether these elements were considered useful or practical by the practitioners, would strengthen the interpretation of your findings.

Have you considered providing rapid diagnostic tests to primary care physicians to support more accurate identification of infections that truly require antibiotic therapy? The use of such tools, such as C-reactive protein testing or rapid streptococcal tests, could enhance diagnostic confidence and reduce unnecessary antibiotic prescribing. Including a brief discussion on whether this was considered, and if not, why, would add value to the manuscript.

**Reviewer**	**2**
**Name**	**Lindbaek, Morten**
**Affiliation**	**University of Oslo**
**Date**	**08-Oct-2025**

COI **None**

Review of paper on antibiotic prescribing in China, qualitative

By Morten Lindbæk, prof em, University of Oslo

General comments:

This is an Important research field, especially in China that globally contributes to a high antibiotic consumption and thus to AMR in the far East. The primary study showed a significant reduction of prescribing.

This qualitative study was well performed and is giving a number of interesting findings. There were mixed views on the usability of the specific parts in the intervention. It is valuable to do this research in rural China as few studies have been done previously

I have some specific questions

1. How high is the prescribing in China compared to other countries, this could be specified as important background data

2. Do you have any data on AMR in China, also important as background?

3. I miss some statements of the preconceptions of the researchers? Did this influence the qualitative data in any ways?

4. Patients' expectations should be discussed more thoroughly, especially the clear difference of age and the willingness to receive information. How to address older people?

5. Peer discussions were problematic, and this is linked to cultural dimensions and differences between countries. These should be referred, to try and explain for example the reluctance to take part in peer discussions. Hofstede's theory on cultural dimensions is relevant as to antibiotic prescribing. Both Uncertainty avoidance and Power distance are closely linked to prescribing. I assume power distance is quite high in China, and this can be part of the explanation of lack of peer group discussions. Ref Hofstede

Refs Hofstede

Deschepper R, Grigoryan L, Lundborg CS, Hofstede G, Cohen J, Kelen GV, Deliens L, Haaijer-Ruskamp FM. Are cultural dimensions relevant for explaining cross-national differences in antibiotic use in Europe? BMC Health Serv Res. 2008 Jun 6;8:123. doi: 10.1186/1472-6963-8-123.

VERSION 1 - AUTHOR RESPONSE

Reviewer: 1

The study is both interesting and relevant. However, I would kindly request the authors to provide more context and explanation regarding the Chinese healthcare system. From a Western perspective, some aspects of the article are difficult to fully understand due to differences in clinical practice and healthcare infrastructure. For instance, it would be helpful to clarify what the "Zhiyi system" refers to, as this term may not be familiar to international readers. Additionally, including information about Chinese clinical guidelines for the management of RTIs would significantly enhance the article's accessibility. Even a brief overview in the supplementary material would be valuable, particularly for common RTIs such as pneumonia, acute exacerbations of COPD, acute bronchitis, pharyngitis, and tonsillitis, since most readers may not be acquainted with the Chinese recommendations for these conditions. Moreover, the concept of using "anti-inflammatory medicines" in place of antibiotics may appear unusual or even concerning to those trained in Western medicine. Further clarification and discussion of this practice, especially regarding its rationale, usage, and supporting evidence in China, would be important for international understanding and comparison.

Thank you for the helpful suggestions regarding the need to provide more context and explanation of the Chinese healthcare system. We have now added this information in several sections of the manuscript to help readers who may not be familiar with the system.

Specifically, we have included a brief introduction to China's rural primary care system and discussed issues related to its rollout period (lines 92–100). As suggested, we have also added information about two national Chinese guidelines for primary care and included them as supplementary documents, and briefly explained how antibiotics are recommended for a range of RTIs. In addition, we have incorporated evidence showing that antibiotic prescribing rates in primary care remain high (lines 82–90).

The "Zhiyi system" is a province-wide digital health platform that supports practitioners in clinical decision-making, electronic health record management, and public health monitoring. We have now provided further details about this system in the section where it is discussed alongside our DSS (lines 494–499).

We have also expanded our explanation on the findings that the term "anti-inflammatory medicines" was usually used by practitioners and patients when referring to antibiotics, which reflects a broader cultural issue linked to the differences between Chinese and Western biomedical models. Specifically, infections are often understood in Chinese medicine as bodily reactions rather than invasions by external pathogens, and antibiotics are viewed as anti-inflammatory agents that "cool" the body. This understanding also influenced the way we drafted messages in our intervention materials using the local term for antibiotics (lines 533–545).

In the Strengths and Limitations sections (both), you mention that a limitation of the study is the lack of direct patient accounts regarding the intervention. However, you also state that patients' views and responses were indirectly captured through practitioner interviews and

informal conversations with fieldworkers from both intervention and control clinics. This remains a supposition; conclusions about patients' perspectives cannot be reliably drawn without directly interviewing the patients themselves. I kindly suggest deleting this latter part for accuracy.

Thank you for the suggestion. We have removed the relevant statements referring to the indirect capture of patients' views from both sections of the manuscript (lines 65-69 & lines 564-566).

Could you please clarify who issued the public letter of commitment to reducing unnecessary antibiotic prescribing, which was provided to health practitioners for sign-off and display in their clinics? Was it issued by local or national health authorities, or by the steering committee of the randomised clinical trial? Additional details would be helpful. I suggest including this information in Box 1 for clarity.

The public letter was designed and issued by the research team to health practitioners. We have now added these details in Box 1.

Regarding the distribution of leaflets, could you please provide more detail on how doctors in the intervention group actually used them? Specifically, was the process limited to simply handing out the leaflets, or did it also involve discussing key points with patients during the consultation? This is an important consideration, as the effectiveness of the intervention largely depends on how the information is communicated and received by patients. As some doctors rightly pointed out, handing out a leaflet alone is of limited value if time is not spent addressing the conflicting information and guiding patients through it.

This intervention component primarily focuses on doctors, aiming to provide them with an alternative message to deliver when antibiotics are not prescribed, thereby reassuring both patients and themselves. Therefore, the research team did not set specific requirements regarding how this communication should be conducted, as it was largely left to the doctors' discretion. This explanation has now been added to Box 1.

Did you have any discussions with doctors about the individual components of the multifaceted intervention? It would have been valuable to engage with frontline doctors beforehand to assess the perceived relevance, feasibility, and acceptability of each of the five components. Based on the information provided in the manuscript, it appears that few doctors actively used the decision support system or participated in the peer support groups. Further explanation of why this was the case, and whether these elements were considered useful or practical by the practitioners, would strengthen the interpretation of your findings.

Yes, we used the Person-Based Approach and involved various frontline practitioners and patients in the discussion, which helped make the intervention more acceptable to practitioners. This information and reference have been added in lines 105-107.

Have you considered providing rapid diagnostic tests to primary care physicians to support more accurate identification of infections that truly require antibiotic therapy? The use of such tools, such as C-reactive protein testing or rapid streptococcal tests, could enhance diagnostic confidence and reduce unnecessary antibiotic prescribing. Including a brief discussion on whether this was considered, and if not, why, would add value to the manuscript.

Thank you for this suggestion, and we have added the discussion focusing on testing in lines 515-520. Blood tests for patients presenting with RTIs are available at the THC level but not at the village clinic level. However, practitioners often misinterpret a high white blood cell count as an indication for antibiotic use, and we have therefore included recommendations on this issue in our training.

Reviewer: 2

General comments:

This is an Important research field, especially in China that globally contributes to a high antibiotic consumption and thus to AMR in the far East. The primary study showed a significant reduction of prescribing.

This qualitative study was well performed and is giving a number of interesting findings. There were mixed views on the usability of the specific parts in the intervention. It is valuable to do this research in rural China as few studies have been done previously.

Thank you for the general comments.

I have some specific questions:

1. How high is the prescribing in China compared to other countries, this could be specified as important background data

Evidence on antibiotic prescribing in China's primary care compared with other countries and the WHO recommendations (lines 78–81) has been added to the introduction section. We have also included data on antibiotic prescribing for RTIs in primary care in China (lines 87–90).

2. Do you have any data on AMR in China, also important as background?

We have added evidence on the burden of AMR (deaths associated with AMR) in China at the beginning of the introduction section (lines 74-76).

3. I miss some statements of the preconceptions of the researchers? Did this influence the qualitative data in any ways?

We have now included some statements on researchers' preconceptions and the strategies used to minimise it at the end of the Methods section (lines 200–205).

4. Patients' expectations should be discussed more thoroughly, especially the clear difference of age and the willingness to receive information. How to address older people?

We have now expanded the paragraph to provide a more thorough discussion of patients' expectations and age-related differences. Evidence suggests that older people tend to have higher expectations for antibiotic prescriptions, which is associated with an increased risk of inappropriate prescribing. In addition, differences in how illness and antibiotics are understood within Chinese and Western biomedical systems influence patients' expectations for treatment. Older adults, who often adhere more closely to the traditional Chinese medical model, may require a more culturally sensitive way to communicate about appropriate antibiotic use. We have discussed several suggestions for engaging older patients through culturally sensitive and visually based communication approaches (lines 524-554).

5. Peer discussions were problematic, and this is linked to cultural dimensions and differences between countries. These should be referred, to try and explain for example the reluctance to take part in peer discussions. Hofstede's theory on cultural dimensions is relevant as to antibiotic prescribing. Both Uncertainty avoidance and Power distance are closely linked to prescribing. I assume power distance is quite high in China, and this can be part of the explanation of lack of peer group discussions. Ref Hofstede

Refs Hofstede

Deschepper R, Grigoryan L, Lundborg CS, Hofstede G, Cohen J, Kelen GV, Deliens L, Haaijer-Ruskamp FM. Are cultural dimensions relevant for explaining cross-national differences in antibiotic use in Europe? BMC Health Serv Res. 2008 Jun 6;8:123. doi: 10.1186/1472-6963-8-123.

Thank you for suggesting Hofstede's theory and the relevant reference. We have now discussed the lack of peer support groups, which is related to the deeply embedded hierarchical structure of China's healthcare system. Practitioners may therefore prefer

leadership-driven decision-making, including in relation to antibiotic prescribing (lines 475-479).

VERSION 2 - REVIEW

**Reviewer**	**2**
**Name**	**Lindbaek, Morten**
**Affiliation**	**University of Oslo**
**Date**	**09-Dec-2025**
**COI**	

I think the paper has improved considerably. The authors have complied with the suggestions from the referees. There have been few papers from China on this important topic that adds to the value of this paper.

One comment - a number of the supplementary is written in Chinese, which I assume the editors will consider.

VERSION 2 - AUTHOR RESPONSE

Reviewer 2 comments

I think the paper has improved considerably. The authors have complied with the suggestions from the referees. There have been few papers from China on this important topic that adds to the value of this paper.
One comment - a number of the supplementary is written in Chinese, which I assume the editors will consider.

Thanks very much. I have now removed these Chinese supplementary documents (two guidelines), and only summarised in the manuscript the relevant issues related to the guidelines. These two guidelines have been cited as references 9 and 10.
